# The Question of Surgical Interference in Cases of Perityphlitis

**Published:** 1892-04-30

**Authors:** 


### SURGERY.

### THE QUESTION OF SURGICAL INTERFERENCE IN CASES OF PERITYPHLITIS.

A young man is attacked with severe pain in the right iliac region, accompanied by vomiting, and there is marked tenderness there, indicating the probable perforation of the vermiform appendix. We know that one of three things may happen, either general peritonitis may come on and prove rapidly fatal; localised peritonitis may be set up, leading to adhesions and the limitation of the disease; or no pus may form, but the appendix may merely get tied down by adhesions. Here we have results of widely different diagnosis, and calling for different treatment, and yet how are we to tell what is going to happen in any given case, whether rapidly fatal and general peritonitis, or the less fatal localised abscess, or the comparatively trivial dry adhesions will result? Of course we are writing now of the very early stage of the affection, and before pus has time to form, and, therefore, before there is any evidence of its presence to help us in the diagnosis—at a stage, in fact, when it is of the utmost importance to do the right thing in the way of treatment, because if our prognosis could be more certain at the very beginning, if we could tell that the case would be a dangerous one, we might deal with the perforation as we would any other perforation into the peritoneum, by suturing the gut, and washing out the peritoneal cavity, and in this case of course removing the perforated appendix. But it is the peculiarity of perforation of the appendix that usually we do not get such alarming symptoms from it as from intestinal or stomach perforations, because, generally, the appendix is so blocked behind the concretion or has so narrow a lumen that fæces do not escape into the peritoneum. There is not the same amount of shock as in perforation in some part of the intestinal canal where there is a ready escape of the contents. Again Treves says that some cases of the severest suppurative form occasionally begin with quite mild symptoms; and on the other hand, that cases that quickly end by the formation of dry adhesions may begin with sudden pain and vomiting with quite acute symptoms. This seems to make the question of prognosis in any given case almost a hopeless one. If, however severe and sudden the pain, the case may yet very probably end in the formation of dry adhesions and recovery, we are not justified in opening the abdomen. Seeing then that we cannot be sure from the severity or character of the symptoms what is going to happen, the question resolves itself into this, What are the chances of such a favourable termination in cases which begin with severe symptoms? and this question can only be answered by the careful recording of a large number of cases with a view to determine the point. First we must remember that the mild form is in many cases unconnected with mischief in the appendix, for it is due to the perforation of stercoral ulcers in the cæcum, *i.e.*, to ulcers due to the irritation caused by the lodgement of fæcal masses there in constipation. This gives us some little help, for we usually get in such cases a history of constipation with the passage of scybala. Unfortunately, this form, like the severe one, usually occurs in young adults, so that age does not help us much, only in so far as more cases occur after 40 than in the severe form. But it is rather to the severity of the symptoms as a whole that we are to look for guidance in these cases. If they are severe the probability is we have to deal with a case which will go on to the formation of pus, and will incur the risk of general peritonitis. Treves* gives the cause of the symptoms in the suppurative, and the non-suppurative form thus. In the case in which we may expect abscess, he says, "the symptoms are more severe and progress more rapidly. There is often an initial chill or rigor. The pain is severe, the vomiting marked, the tenderness and other signs of peritonitis are pronounced. The fever is usually high." This applies to the commencement, except possibly the fever. The absence in these suppurative cases of the early swelling present in those where the cæcum is blocked with scybala in the non-suppurating cases, due to perforation of stercoral ulcers is to be noted. The swelling due to pus forms later, and, unlike the scybalous swelling, is fixed, and can often be made out through the rectum. In the suppurative form, too, "the pain is apt to radiate, to the testes, thigh, or perineum, and to be associated with disturbances of micturition and tenesmus. Pain on moving the right thigh is often marked." These symptoms, we presume, are also late symptoms, after pus has begun to form. "The local swelling or the area of dulness take on the phenomena attending suppuration, and at a varying period from the commencement of the trouble evidences of pus are distinctly felt."

* *British Medical Journal*, November 9th, 1889.

Treves describes the non-suppurative form thus: "The pain appears suddenly, but is on the whole less severe than in the graver form. There is seldom any rigor, the fever is not so high, the vomiting not so marked, and the pain is less apt to radiate to distant parts, for example, to the thigh or testes. The tenderness is, perhaps, less pronounced, the tumour appears earlier—possibly from the first—it is comparatively large, is apt to be doughy, to feel less fixed; it cannot so readily be made out through the rectum. Bladder troubles are usually absent."

Occasionally, perforation of the appendix does produce at once not a local but a general peritonitis, so rapidly fatal that persons have died from it in forty-eight hours. Here, of course, our duty is plain—we have no reason to delay laparotomy, and, therefore, we need not consider such cases further.

But at the commencement of the other forms of the disease, seeing that so many cases recover without even the formation of abscess—so large a number that several physicians regard perityphlitis as a disease in which surgical treatment is quite unnecessary, as they think the cases tend to get well themselves with opium and rest—we cannot think that we are justified in opening the abdomen when we are so far from able to prognosticate whether or not pus will form or the disease quickly subside without. It is true that some cases die very quickly from early rupture of such a purulent collection, but, as Treves points out, we should hardly be right in operating in every case directly symptoms egan, seeing so many cases recover which begin with quite severe symptoms. And even if abscess forms only eight out of sixty-seven cases collected by Bull ruptured into the peritoneum. But although, therefore, we are not justified in performing laparotomy before there are signs of a collection of pus, as soon as this can be made out (and a rectal examination should always be made) then we should at once evacuate it. This seems to be the general opinion of surgeons. If we wait it may rupture into the general peritoneal cavity at any moment. This evacuation should be made by an incision outside the abscess, *i.e.*, between it and the anterior spine so as to get at the pus without getting into the general peritoneal cavity.

Bull* advocates early abdominal section without waiting for local signs of pus. He says "the risk of operation is less than the risk of waiting," and "that the more rapid the development of the symptoms the earlier should the surgeon interfere." But as we have pointed out, many cases beginning quite acutely recover. Are we then to run the risk of an abdominal operation in all cases? Again, when a purulent collection can be made out the parts around have probably become adherent to the abdominal wall, and we can safely evacuate the pus (by the incision just described) without letting the pus into the peritoneal cavity; but if we open the abdomen very early in the case we may certainly find a collection of pus shut in by adherent coils of gut, but in order to evacuate it we may have to let the pus first into the general peritoneal cavity, pus perhaps as fœtid as fæces, because sufficient time had not elapsed for the intestines around to become adherent to the abdominal wall.

There is one method of exploration for a purulent collection in the abdomen which we have not so far mentioned, and that is by the exploring syringe, or aspirator. Bull strongly recommends its use, Treves as strongly condemns it. Its advantages are obvious, its risks are puncture of a large vessel, or the ureter, or first the bowel and then perhaps the cellular tissue outside, inoculating fæcal matter there. In several cases the exploring needle has not detected pus which has been evacuated by incision directly afterwards.

